# A Chess and Card Room-Induced COVID-19 Outbreak and Its Agent-Based Simulation in Yangzhou, China

**DOI:** 10.3389/fpubh.2022.915716

**Published:** 2022-06-17

**Authors:** Shijing Shen, Wenning Li, Hua Wei, Lin Zhao, Runze Ye, Ke Ma, Peng Xiao, Na Jia, Jieping Zhou, Xiaoming Cui, Jianhua Gong, Wuchun Cao

**Affiliations:** ^1^State Key Laboratory of Pathogen and Biosecurity, Beijing Institute of Microbiology and Epidemiology, Beijing, China; ^2^School of Public Health and Health Management, Gannan Medical University, Ganzhou, China; ^3^National Engineering Research Center for Geoinformatics, Aerospace Information Research Institute, Chinese Academy of Sciences, Beijing, China; ^4^University of Chinese Academy of Sciences, Beijing, China; ^5^Institute of EcoHealth, School of Public Health, Cheeloo College of Medicine, Shandong University, Jinan, China

**Keywords:** COVID-19, SARS-CoV-2 Delta variant, agent-based model, SEIR model, scenario simulation

## Abstract

**Objective:**

To evaluate epidemiological characteristics of the COVID-19 outbreak that resurged in Yangzhou and to simulate the impact of different control measures at different regional scales.

**Methods:**

We collected personal information from 570 laboratory-confirmed cases in Yangzhou from 28 July to 26 August 2021, and built a modified susceptible-exposed-infected-removed (SEIR) model and an agent-based model.

**Results:**

The SEIR model showed that for passengers from medium-high risk areas, pre-travel nucleic acid testing within 3 days could limit the total number of infected people in Yangzhou to 50; among elderly persons, a 60% increase in vaccination rates could reduce the estimated infections by 253. The agent-based model showed that when the population density of the chess and card room dropped by 40%, the number of infected people would decrease by 54 within 7 days. A ventilation increase in the chess and card room from 25 to 50% could reduce the total number of infections by 33 within 7 days; increasing the ventilation from 25 to 75% could reduce the total number of infections by 63 within 7 days.

**Conclusions:**

The SEIR model and agent-based model were used to simulate the impact of different control measures at different regional scales successfully. It is possible to provide references for epidemic prevention and control work.

## Introduction

The COVID-19 pandemic has been ongoing since 2020. As of 7 November 2021, there were 248, 467, 363 cases of SARS-CoV-2 virus infection, including 5,027,183 that resulted in death all over the world as reported by the World Health Organization (1). According to the American Centers for Disease Control and Prevention, there have been eight viral variants: Alpha, Beta, Gamma, Delta, Epsilon, Iota, Eta, and Kappa (2).

On 28 July 2021, there was a COVID-19 outbreak in Yangzhou, Jiangsu Province, and the chess and card room became the focus. This outbreak was caused by the Delta variant and spread rapidly, due to delayed nucleic acid testing and lack of vaccination in the first confirmed case. The chess and card room, as the focus point, had a large population density and poor ventilation. The epidemic, which infected 570 people and took 28 days to bring under control, had a severe impact on social and economic development.

The development of the epidemic can be divided into two regional scales: a large-scale environment and small-scale environment. How to contain the spread of the epidemic and return to normal life in a large-scale environment through nucleic acid testing and vaccination and how to control population density and increase ventilation to ensure orderly life in a small-scale environment are the urgent problems to be solved.

There are many models for simulating large-scale environments. Giulia Giordano and his collaborators made an SIDARTHE model, to simulate restrictive social-distancing measures (3). Cui and his partner build an SEIR model to evaluate the effect of different non-pharmaceutical interventions (NPIs) in Beijing (4). Prem et al. (5) used an age-structured SEIR model to assess the impact of physical isolation on the development of COVID-19 in Wuhan. However, they all used the traditional SEIR model to simulate the initial spread of COVID-19, which did not consider emerging influencing factors such as vaccination coverage and nucleic acid testing.

For COVID-19 simulation in a small-scale environment, the agent-based model was selected. The agent-based model can describe the attributes and behaviors of each individual, considering the spatial structure relations at the individual level. Traditional models cannot evaluate the effect of individual attributes and spatial structure on disease transmission. In consideration of this point, the agent-based model is suitable for a study in a small-scale environment (6).

Therefore, to further analyze the effect of epidemic prevention and control, we collected the information of 570 confirmed cases in Yangzhou and constructed the improved SEIR model for the large-scale environment and the agent-based model for the small-scale environment to simulate the epidemic control effect after implementing different measures.

## Methods

### Data Collection

All information about confirmed cases in Yangzhou was collected from Yangzhou Announcement, Health Yangzhou, and the Yangzhou Health Commission. We extracted information about demographic characteristics (age, gender, residence location, visited location), exposure history, close contacts, clinical type, key time points (nucleic acid test date, quarantined date, and diagnosed date), and travel path of confirmed cases from 28 July to 26 August 2021.

Data for targeted interventions were obtained from the National Health Commission of the People's Republic of China and the Yangzhou Health Commission. The population data came from the seventh national census of China. The permanent resident population of Yangzhou was 4,559,797, including 542,305 in Guangling District, 726,906 in Hanjiang District, and 926,577 in Jiangdu District.

The aggregation of natural blocks in Yangzhou city was collected from the open data platform. The point of interest (POI) data of Yangzhou city included district and county, administrative division code, longitude, latitude, name, type, telephone number, and address, which were extracted and integrated through ArcGIS V.10.2 software (ESRI, Redlands, California, USA).

**Figure 1:**
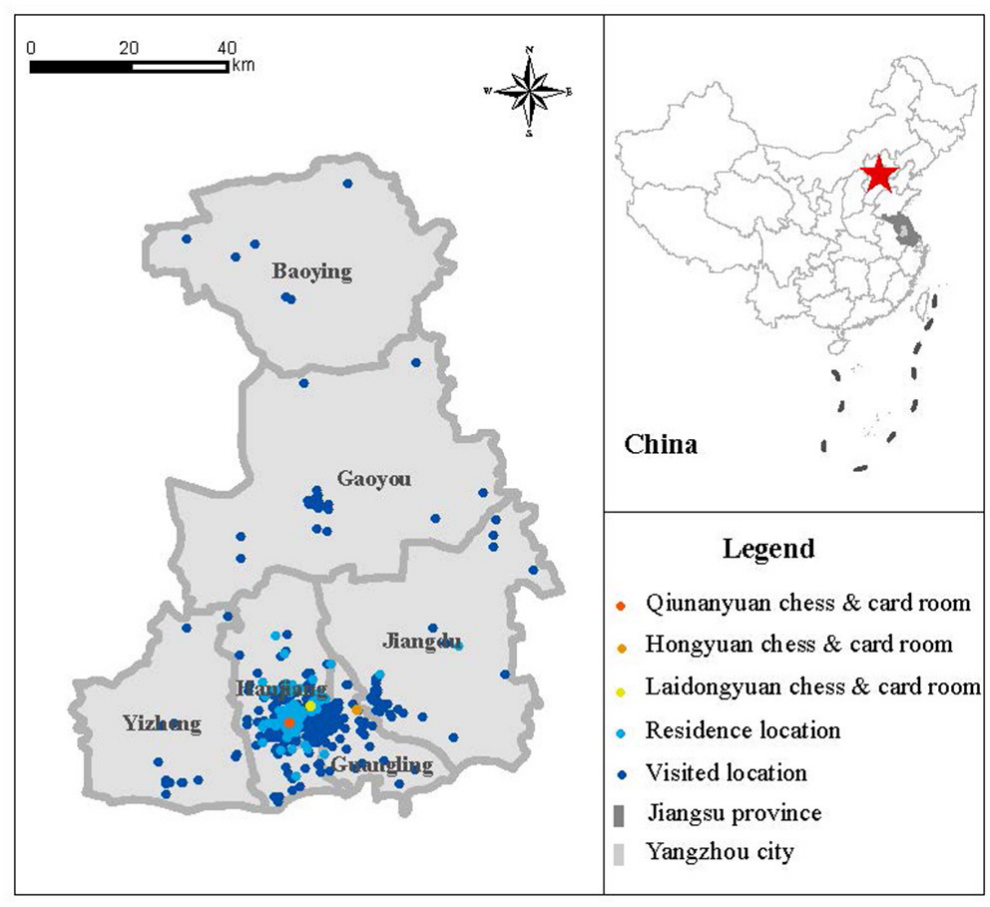
The residence location and visited location of confirmed cases (Yangzhou, China, 2021).

We also extracted the geographic location information of 570 confirmed cases based on administrative divisions and street networks in Yangzhou. The geographic distribution map produced by ArcGIS V.10.2 software (ESRI, Redlands, California, USA) showed the residence location of confirmed cases, visited locations of confirmed cases, and the location of the chess and card room in Yangzhou (Figure 1). According to the distance between the residential location of the case and the chess and card room, the effectiveness of the control measures adopted in Yangzhou was analyzed.

### Simulation Model

#### Simulation of Yangzhou

We adopt an improved SEIR model to investigate different strategies and measures in Yangzhou. This model divided the population into five compartments, i.e., the vaccinated population V, the susceptible population S, the exposed population E, the infected population I, and the removed population R. The removed population contained individuals removed from transmission due to death or recovery, as well as individuals that were quarantined. Once the infected persons obtained laboratory confirmation, they would be quarantined and would not cause secondary infection anymore (7).

In this model, the age of cases was divided into three parts (younger than 15, 15 to 59, and older than 59) (Supplementary Table 1). The infection rate and incubation period were characteristic parameters of the disease, which were determined by the characteristics of the pathogen itself. The optimal distribution of these parameters was calculated by case data of epidemiological investigation. Nucleic acid detection rate and vaccination rate were obtained by calculating case data or consulting published literature.

Considering the characteristic parameters of COVID-19 and non-pharmaceutical interventions, we used an improved SEIR model to simulate the effect of vaccination rate and a nucleic acid test on containing the spread of the epidemic. We simulated two hypotheses: (1) increasing vaccination coverage among the elderly and (2) showing negative nucleic acid test within 48 h before traveling.

The process of modeling was mainly divided into three parts: (1) population division: people were divided into three groups by age: children (0–14 years old), adults (15–59), and the elderly (over 60); (2) parameter setting: simulating parameters such as the incubation period and transmission rate to fit the evolution of diseases; and (3) strategies assessment: closing recreational places, quarantines in homes, restrictions on individual movement, and nucleic acid testing.

Any individual could be in one of five different states (infectious or healthy): vaccinated (V), susceptible (S), exposed (E), infected (I), and removed (R). Removed included being quarantined, recovered, and died. Vaccination was added to the improved model to assess how vaccines, age, nucleic acid testing, and social isolation policies affect the evolution of the infectious disease. Supplementary Figure 1 shows the transition dynamics of infectious diseases at each level of the model.

#### Simulation of Chess and Card Room

The initial outbreak was started in the chess and card room –a place for entertainment. To understand the transmission process of the virus in indoor places clearly, we took the Qiunanyuan chess and card room, a major transmission site in Yangzhou, as our research object to simulate the transmission of the virus based on the known epidemiological characteristics of the COVID-19 virus.

Agent-based modeling uses computer technology and artificial control logic and rules to simulate individual decisions and behaviors, which interact with the environment and other subjects while continuously developing and evolving (8). It can be used to study the impact of individual attribute behavior on epidemic spread (9–11). The Monte Carlo algorithm is an algorithm for computers that is used to simulate the behavior of other systems. It is not an exact method, but a heuristic one, typically using randomness and statistics to get a result (12).

By setting rules of interactive infection and state transition between agents, an agent-based simulation system of COVID-19 transmission in the indoor scene of the chess and card room was established to simulate the target selection, walking, avoiding, and playing behaviors of guests in the chess and card room, and to reveal the change process of virus transmission in indoor chess and card rooms over time. Multi-scene simulation experiments were carried out under the conditions of different ventilation and agent density parameters.

The model had four main parts: (1) the spatial structure where agents moved in: the area of the chess and card room was about 100 square meters and was divided into three areas, including a tea room, toilet, and recreational area with 10 tables (Supplementary Figure 2). (2) The flow of crowd activities in the chess and card room and home. The main parameters were the frequency to the chess room and the time staying in the chess room of the agent (Supplementary Figure 3). There were 74 infected patients who had visited the Qiunanyuan chess and card room in Yangzhou. We constructed the distribution N~(4.32, 2.3) of the entry frequency of agents and the distribution N~(3, 1) (h) of entertainment time based on their data in the chess and card room 7 days before the emergency mechanism was launched. (3) The rules of agent infection: the main consideration for agent infection was indoor ventilation and the distance between close contacts. To simplify the state transition rules of customer agents in the chess and card room, we divided them into two states: susceptible and exposed. (4) The infected status of the agent.

## Results

### Situation of the Epidemic in Yangzhou

In this COVID-19 epidemic, there were 570 laboratory-confirmed cases in Yangzhou up to 26 August 2021. The characteristics of the cases are shown in Table 1. The proportion of women (57.9%) was higher than men (42.1%). The median age of these cases was 54, in which the inter-quartile range (IQR) is between 33 and 68, with 59 juveniles (10.4%), 280 adults (49.1%), and 231 elderly people (40.5%). The severity of most cases was moderate (67.7%), followed by mild (30.7%) and severe (1.6%). All severe cases were over 60 years old. None of the total 566 cases were critically severe. During this COVID-19 epidemic in Yangzhou, 115 (20.2%) of the 566 cases were exposed in the chess and card room, and others were infected by those who had been in that place. The transmission chain (Supplementary Figure 4) revealed that the first case infected some people in the chess and card room and then those people infected others. Besides, the travel path of cases was around the card room, indicating that the chess and card room was an important point in this epidemic.

**Table 1:** Characteristics of cases in Yangzhou from 28 July to 26 August 2021 (Laboratory-confirmed cases, China, 2021).

**Variables**	**Median (IQR) /*n* (%)**
Gender	
Male	240 (42.1%)
Female	330 (57.9%)
Age	54 (33–68)
≤ 14	59 (10.4%)
15–59	280 (49.1%)
≥60	231 (40.5%)
Severity	
Mild	175 (30.7%)
Moderate	386 (67.7%)
Severe	9 (1.6%)
Critically severe	0
Exposed in chess and card room	
Yes	115 (20.2%)
No	455 (79.8%)

### Model

#### Simulation of Yangzhou

Some scholars have carried out relevant studies on vaccination (13) and proposed an improved SEIR model based on the SEIR model to study the impact of vaccination on epidemic spread (14) and optimize distribution strategies (15). Changes in control strategies during the epidemic were direct factors leading to changes in the epidemic curve. Considering the real situation, we set three major strategies: closing places, home quarantine, and local travel restrictions. The number of contacts and nucleic acid detection rate changed with control strategies.

**Figure 2:**
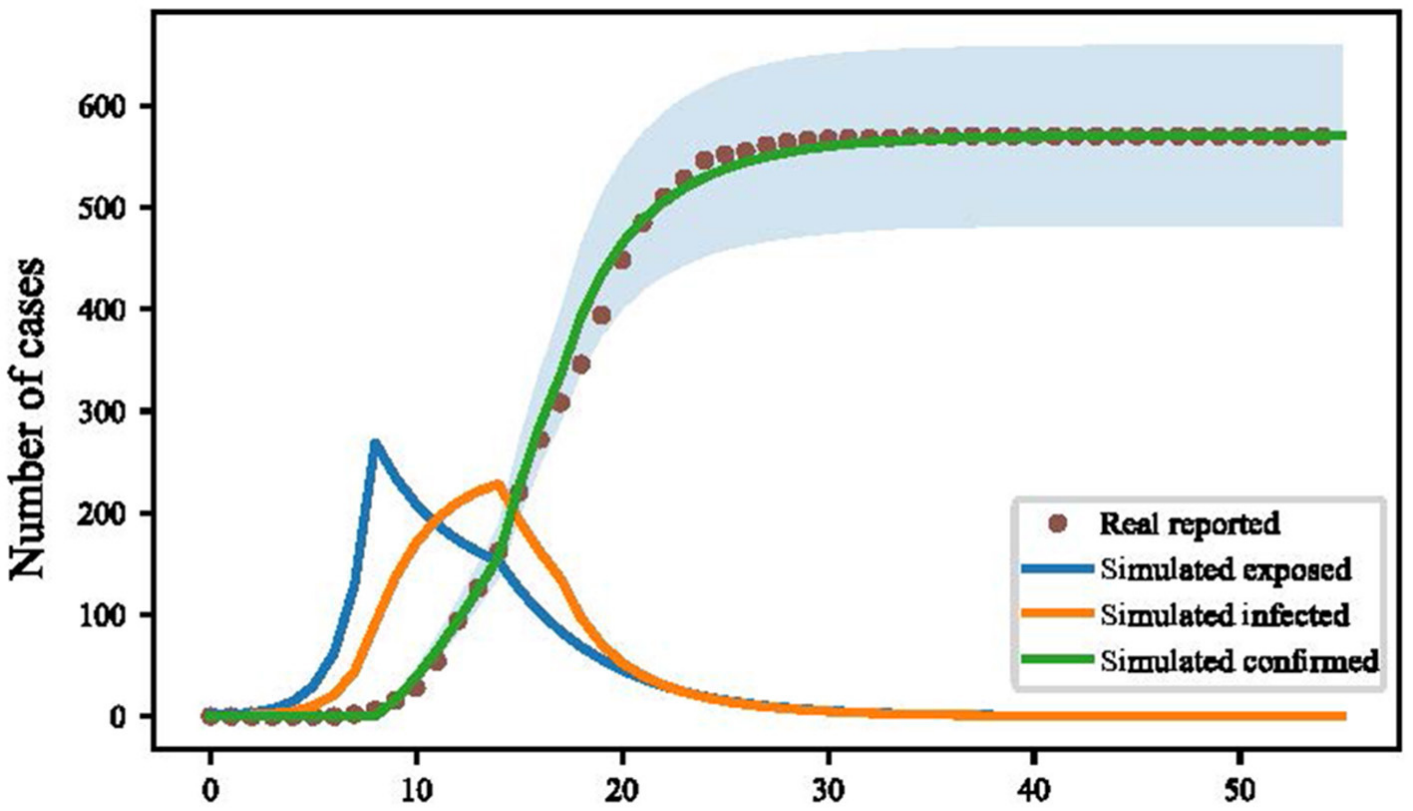
Simulation of cumulative confirmed cases in Yangzhou by improved susceptible-exposed-infected-removed (SEIR) model (Yangzhou, China. 2021).

We used the grid-search method to fit the optimal solution of the model, and the estimated parameters are shown in Supplementary Table 1. We compared the simulation of our model with the actual situation of the epidemic in Yangzhou from 21 July to 21 August 2021 (Figure 2). The determination coefficient R^2^ of the fitting results was 0.998, and the root mean square error (RMSE) was 10.83, showing that our model had an accurate simulation.

Many vaccines have been shown to provide strong protection against COVID-19 (16). We found the majority of cases in this epidemic in Yangzhou were over 60 years old, and the low vaccination rate among this age group might be a factor in the spread of the disease. Based on this situation, we increased the vaccination rate of the elderly to simulate how it impacts the development of the epidemic. Two scenarios were set to simulate the impact of the vaccination rate of the elderly on virus transmission. Details were as follows:

Scenario 1: Increased the vaccination rate of the elderly to 30%.
Scenario 2: Increased the vaccination rate of the elderly to 60%.

**Figure 3:**
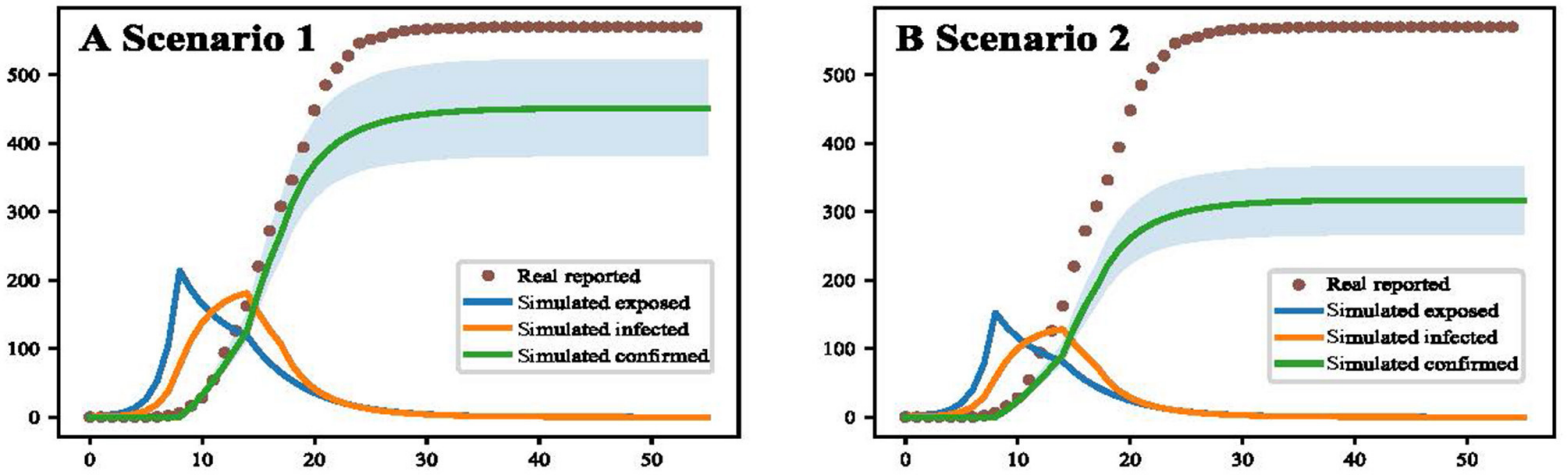
Simulation results of improving vaccination coverage in the elderly (Yangzhou, China, 2021). **(A)** The result of increasing vaccination coverage to 30 % for people over 60 years. **(B)** The result of increasing vaccination coverage to 60 % for people over 60 years.

When the vaccination rate was raised to 30%, the total number of infected people in Yangzhou would be reduced by 119, and when the vaccination rate of people over 60 years old was raised to 60%, the total number of infected people would be reduced by 253 (Figure 3).

**Figure 4:**
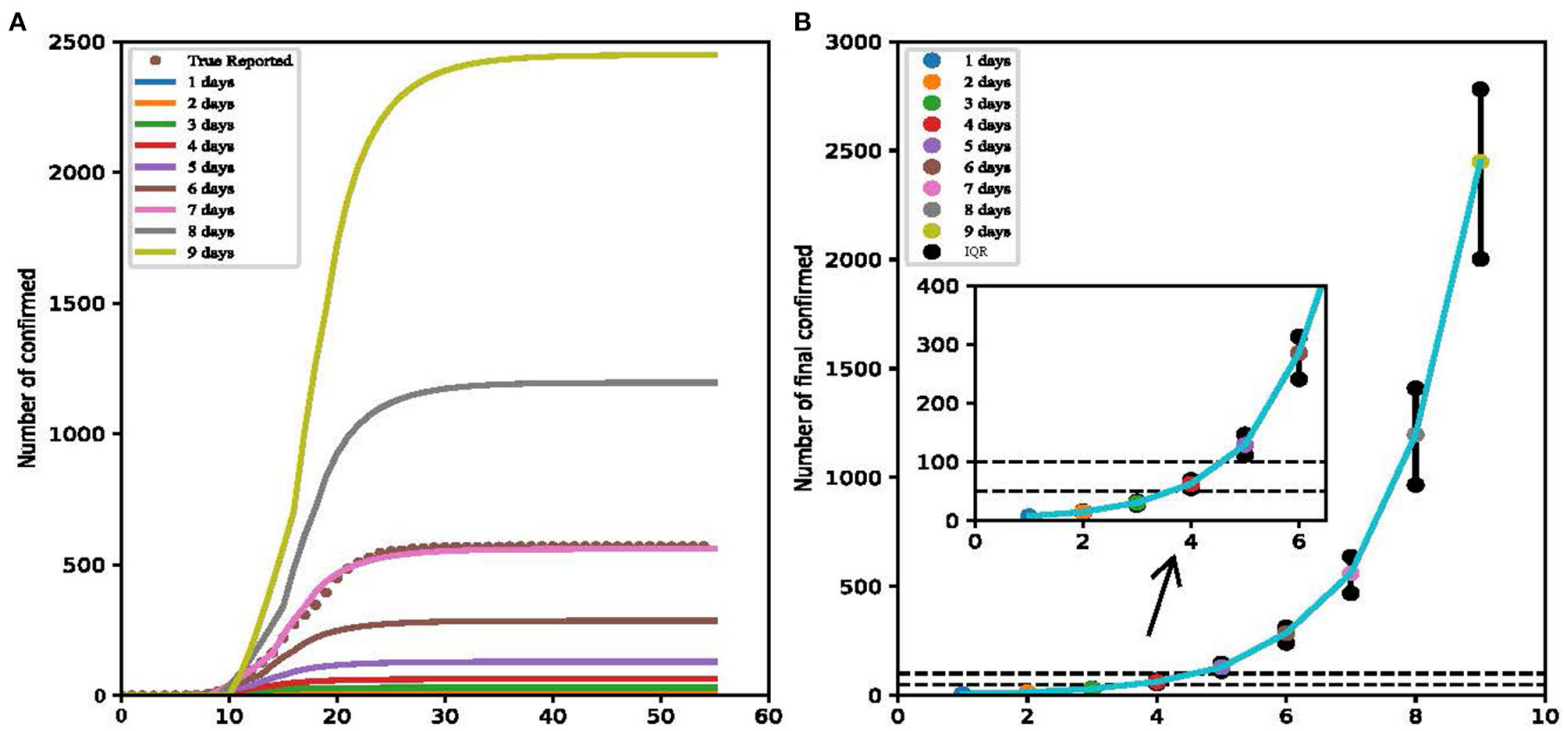
Result of detection intervals from 1 to 9 days (Yangzhou, China, 2021). **(A)** The cumulate infection of nine intervals. **(B)** The total infection number of nine simulations.

In the context of the epidemic, pre-travel nucleic acid detection was considered to be one of the effective strategies to control transmission, for the efficient and reasonable organization of medical resources of nucleic acid testing could find the virus infection quickly (17). We simulated nine detection intervals from 1 to 9 days, finding that the shorter the interval between nucleic acid test and travel date, the better the test strategy effected (Figure 4). The cumulate infection rate of those nine intervals are as follows: 7.0 (IQR: 6.4, 7.4), 14.9 (IQR: 13.8, 15.9), 30.4 (IQR: 26.8, 33.0), 62.5 (IQR: 54.9, 69.6), 129.0 (IQR: 111.4, 146.8), 285.4 (IQR: 240.6, 313.3), 560.0 (IQR: 468.5, 636.4), 1,196.1 (IQR: 965.5, 1,409.7), and 2,448.4 (IQR: 2,002.3, 2,780.9). So based on the simulation results, daily nucleic acid testing had the best effect. In our model, the effect of daily nucleic acid testing on people from medium-high risk areas in Yangzhou could minimize the number of people infected. Pre-travel nucleic acid testing within 3 days could limit the total number of infected people in Yangzhou to 50. Pre-travel nucleic acid testing within 5 days could limit the total number of infected people in Yangzhou to 100.

#### Simulation of Chess and Card Room

**Figure 5:**
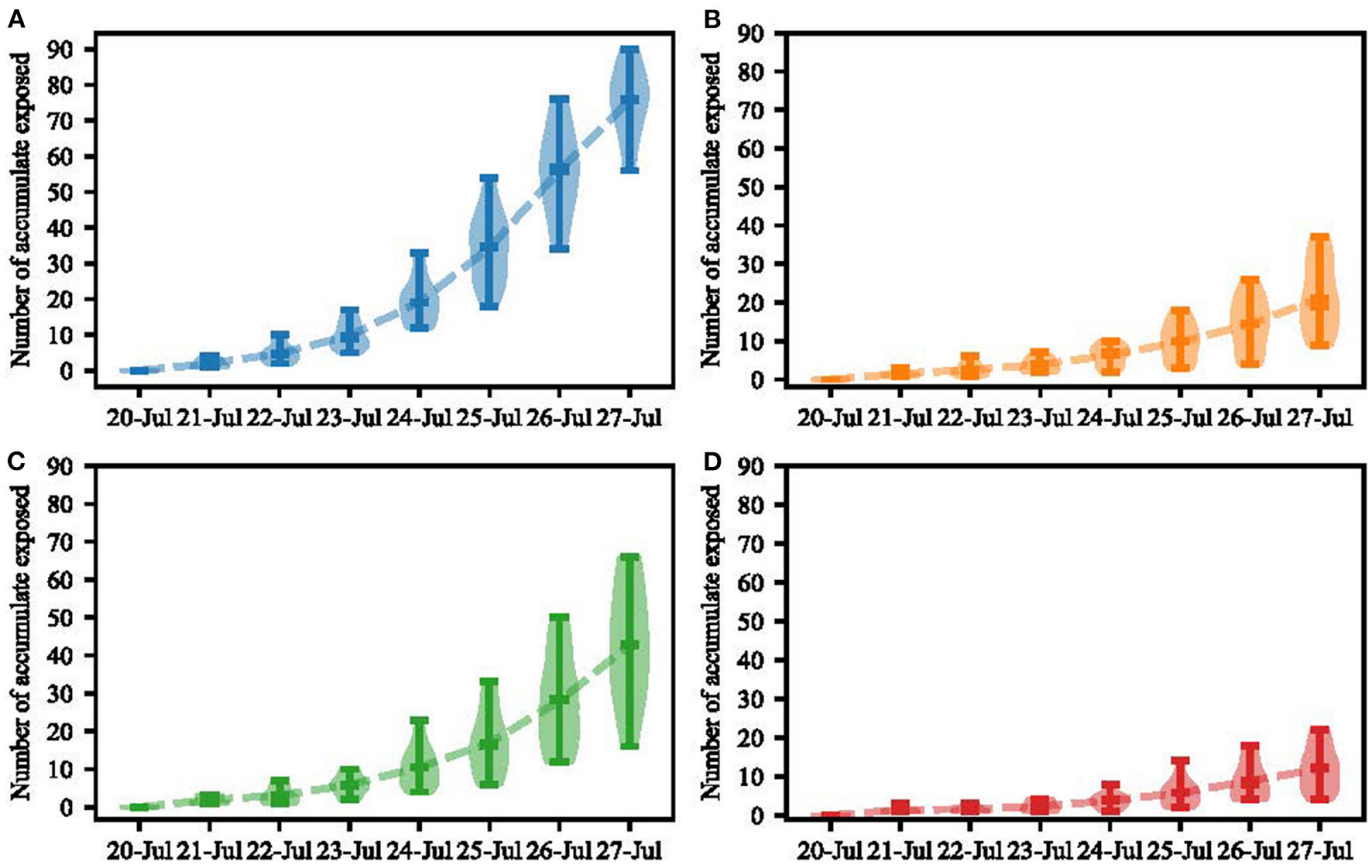
Agent-based model simulation results (Yangzhou, China, 2021). **(A)** Simulation of virus transmission in a chess and card room over an 8-day period. **(B)** Simulation result of scenario 1. **(C)** Simulation result of scenario 2. **(D)** Simulation result of scenario 3.

The agent-based model of the chess and card room was constructed, with a total area of about 100 square meters. We set the transmission probability after long-term contact as 54%, the indoor ventilation rate as 25%, and effective infection distance as 5 m. Then we changed contact intensity in a certain proportion. The Monte Carlo algorithm was used to fit the best result (Figure 5).

The fitting result in our model showed that 75.07 (IQR: 69.0–82.5) persons would be infected in the chess and card room. Three scenarios were set to simulate the impact of indoor population density and ventilation volume on virus transmission. Details were as follows:

Scenario 1: Changing the layout of the chess and card room. Remove mahjong tables 2, 4, 6, and 8. In this scenario, the per capita area was 4.16 square meters. There would be 21 people infected in 7 days (IQR: 13.5–30.5).
Scenario 2: Ventilation volume increased from 25 to 50%. A total of 42 people would be infected in the chess and card room in 7 days (IQR: 31.5–54.0).
Scenario 3: Ventilation volume increased from 25 to 75%. There would be 12 people infected in the chess and card room in 7 days (IQR: 7.0–16.0).

The agent-based model showed that when the population density of the chess and card room dropped by 40%, the total number of infections would reduce by 54 within 7 days. Ventilation increases in the chess and card room from 25 to 50% could reduce the total number of infections by 33 within 7 days. A ventilation increase in the chess and card room from 25 to 75% would reduce the total number of infections by 63 within 7 days.

## Discussion

There were 330 (58%) female cases in this COVID-19 epidemic in Yangzhou, more than male cases (240, 42%). The reason might be related to the fact that going to the chess and card room was a hobby most older women had. In the first case in Yangzhou, a 64-year-old woman came to Yangzhou without nucleic acid negative results and visited her sister. These two infected women then went to chess and card rooms and others in those rooms were infected by her. In the early period, the elderly accounted for the largest proportion of those gathering in the chess and card room (115 cases were exposed in the chess and card room). After that, they went home or to other places such as markets and houses of their relatives, resulting in the infection of adults and juveniles, so the proportion of the elderly was similar to that of others later.

There were nine severe cases during this epidemic and that was a small percentage. Every one of them was older than 60 without vaccination, which had a similar situation to an epidemic in Guangdong (18), indicating that we should pay attention to the vaccine rate in the elderly. Thus, we increased the vaccine rate in the elderly in our model and found this strategy could reduce the final number of infections. So, we advise expanding the limit of vaccine strategy on age here. There is a need to vaccinate healthy older people.

Data fitting according to the SEIR model showed that the probability of susceptible people being infected by infected people was 0.31, while the probability of susceptible people being infected by exposed people was 0.22. We inferred that the reason why infected people had a greater ability to spread disease than exposed people might be the higher viral load infected people had. However, a study with an opposite conclusion shows that the rate of transmission from exposed to susceptible people is 5-fold that of infected to susceptible people (19). This is because they assume that infected people with symptoms will be quarantined, and exposed people will also have normal contact. The incubation our model fitted is 4 days, which is similar to Bai's research and Zhang's real-world study in Guangzhou (20, 21).

The effective rate of being vaccinated was 50% in our model, similar to Zhong's research of a test-negative case-control study in Guangdong, China (22). Besides, the vaccine effectiveness (VE) is 66% during the months when Delta became predominant in some US locations (23). This difference shows that the VE against the Delta variant might be between 50 and 60%, lower than Alpha (14), suggesting that the ability of the vaccine in preventing the Delta variant may have a moderate reduction.

Although the shorter the interval between nucleic acid testing and the travel date, the better the test strategy effects in our model, nucleic acid testing every day will bring a huge medical and economic burden and is not flexible for travel arrangements. Given that the density of population in China and nucleic acid testing of travelers from medium-high risk areas within 3 days could keep the number of infectious people under 100, we believe that 3-day nucleic acid testing might be a suitable strategy for controlling the disease.

In the agent-based model of the chess and card room, we increased the ventilation and effective contact distance to assess their effect, finding that both are effective strategies to control transmission in indoor places like chess and card rooms. A 25% increase in ventilation reduced the number of infections by more than half. Thus, we suggest that ventilation and the distance between the seats should be taken into account when setting up a chess and card room. According to our model and the real situation, if the chess and card room has more ventilation and a larger seat distance, the cases infected in there would be fewer, which can decrease a large account of cases totally. So other small-scale environments can be used as a reference for policy formulation.

This study had some limitations. First, the accurate distribution of chess and card rooms is complex, so we artificially simplified the structure. Second, the activities of agents can be more detailed and intellective. Third, our model assumes only a few major measures in Yangzhou. Finally, we assumed that the compliance of the public was 100% in our model, which is difficult to achieve in reality.

In this study, we constructed an SEIR model and agent-based model to simulate the impact of different control measures at different regional scales, providing reference suggestions for epidemic prevention and control at different scales.

## Data Availability Statement

Publicly available datasets were analyzed in this study. This data can be found at: https://weibo.com/yzfabu.

## Author Contributions

SS, WL, and HW have made a substantial, direct, and intellectual contribution to the work. All authors listed approved it for publication.

## Funding

This study was funded by the Natural Science Foundation of China (81621005), State Key Research Development Program of China (2020YFC0861500), the Strategic Priority Research Program of the Chinese Academy of Sciences, China (XDA19090114), and National Key Research and Development Program of China (2019YFC1200505), and Cheeloo Young Scholar Program of Shandong University.

## Conflict of Interest

The authors declare that the research was conducted in the absence of any commercial or financial relationships that could be construed as a potential conflict of interest.

## Publisher's Note

All claims expressed in this article are solely those of the authors and do not necessarily represent those of their affiliated organizations, or those of the publisher, the editors and the reviewers. Any product that may be evaluated in this article, or claim that may be made by its manufacturer, is not guaranteed or endorsed by the publisher.
