# Supplementary material for: A Chess and Card Room-Induced COVID-19 Outbreak and Its Agent-Based Simulation in Yangzhou, China
Source: Front Public Health. 2022 Jun 17;10:915716. doi: 10.3389/fpubh.2022.915716 (PMC9247329; doi:10.3389/fpubh.2022.915716)
Supplement: Supplementary file 1 [file Table_1.pdf]

A Chess & Card room-induced COVID-19 Outbreak and its Agent-based Simulation in Yangzhou, China

Table S1: The parameters of improved SEIR model

Symbol	Description	Value	Note
N	total population	4559797	From Yangzhou municipal people's government[10]
Child	(<14)	527419	
Adult	(15-59)	2846179	
Older	(>60)	1186199	
input	the number of input cases	1	2021/7/21, Input 1 case from Nanjing, China
α_I	Susceptible being infected by Infected	0.31065	Analog value*
α_E	Susceptible being infected by Exposed	0.220997	Analog value*
β	conversion rate from Exposed to Infected	1/4	Analog value*
θ_1	nucleic acid detection rate	1/7	Close places to home isolation period
θ_2	nucleic acid detection rate	1/3	home isolation to lockdown period
θ_3	nucleic acid detection rate	1/2	lockdown period
ω_1	vaccination rates of children	65%	Simulation results have no official validity
ω_2	vaccination rate of adults	75%	
ω_3	vaccination rate of the elderly	10%	
e	immunization rate of vaccine	50%	Analog value

* The best-fit value obtained by grid search
