# Supplementary material for: A Chess and Card Room-Induced COVID-19 Outbreak and Its Agent-Based Simulation in Yangzhou, China
Source: Front Public Health. 2022 Jun 17;10:915716. doi: 10.3389/fpubh.2022.915716 (PMC9247329; doi:10.3389/fpubh.2022.915716)

A Chess & Card room-induced COVID-19 Outbreak and its Agent-based Simulation in Yangzhou, China

Figure S1: Improves susceptible-exposed-infected-removed model diagram (Yangzhou, China. 2021)

Vaccinated (V), Susceptible (S), Exposed (E), Infected (I), and Removed (R). The probability of being infected is defined based on age, α indicating infection rate of Susceptible; ω indicating vaccination rates, β indicating the conversion rate from Exposed to Infected; θ indicating nucleic acid detection rate; e immunization rate of vaccine; ◷ indicating that an individual must remain in that state for a period of time before changing to another state.

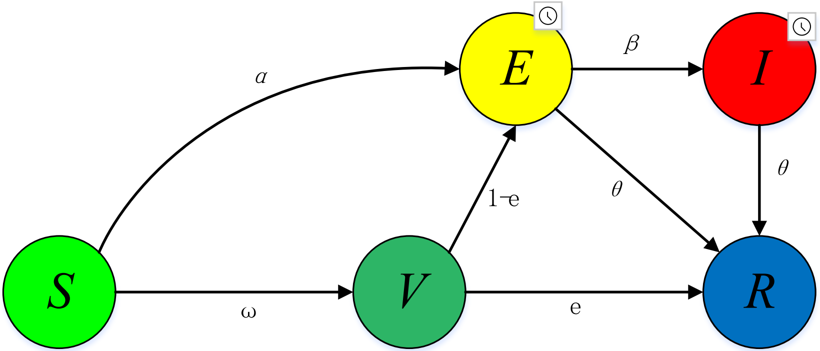

Supplement: Supplementary file 2 [file Image_1.pdf]
