# Supplementary material for: A Chess and Card Room-Induced COVID-19 Outbreak and Its Agent-Based Simulation in Yangzhou, China
Source: Front Public Health. 2022 Jun 17;10:915716. doi: 10.3389/fpubh.2022.915716 (PMC9247329; doi:10.3389/fpubh.2022.915716)

A Chess & Card room-induced COVID-19 Outbreak and its Agent-based Simulation in Yangzhou, China

Figure S2: Layout diagram of chess & cards room (Yangzhou, China. 2021)

The square shape indicates the mahjong table. The asterisk indicates the matching seats of mahjong table.

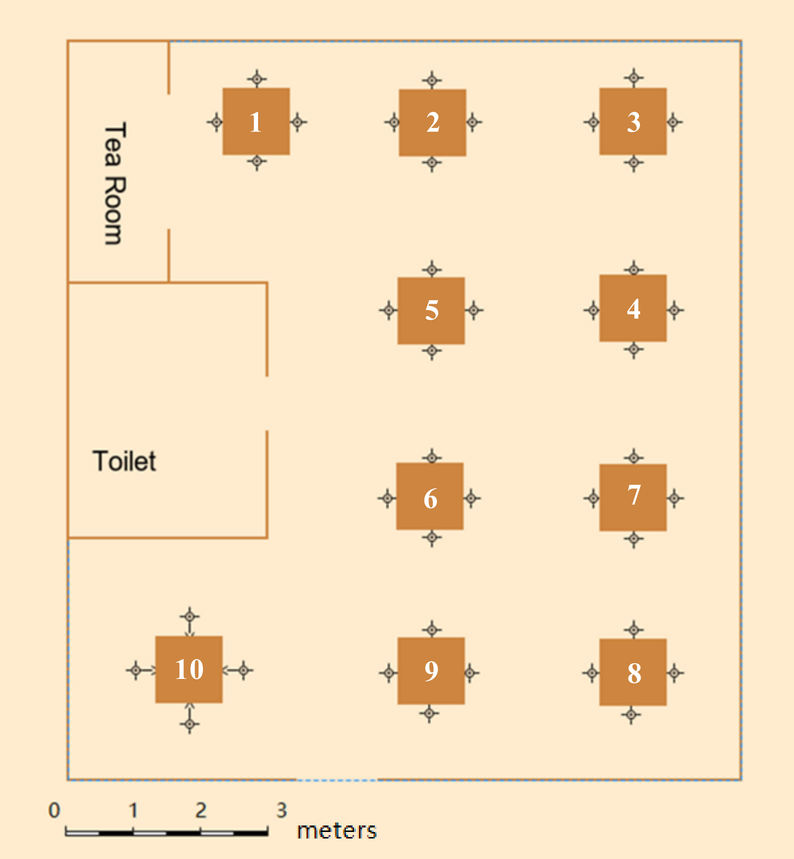

Supplement: Supplementary file 3 [file Image_2.pdf]
